# Why do we climb mountains? An exploration of features of behavioural addiction in mountaineering and the association with stress-related psychiatric disorders

**DOI:** 10.1007/s00406-022-01476-8

**Published:** 2022-08-18

**Authors:** Leonie Habelt, Georg Kemmler, Michaela Defrancesco, Bianca Spanier, Peter Henningsen, Martin Halle, Barbara Sperner-Unterweger, Katharina Hüfner

**Affiliations:** 1 Department of Psychiatry, Psychotherapy, Psychosomatics, and Medical Psychology, University Hospital of Psychiatry II, Medical University of Innsbruck, Anichstrasse 35, Innsbruck, Austria; 2 Department of Prevention, Rehabilitation and Sports Medicine, Klinikum Rechts der Isar, School of Medicine, Technical University of Munich, Munich, Germany; 3 Department of Psychiatry, Psychotherapy, Psychosomatics, and Medical Psychology, University Hospital of Psychiatry I, Medical University of Innsbruck, Innsbruck, Austria; 4 Department of Psychosomatics, Klinikum Rechts der Isar, School of Medicine, Technical University of Munich, Munich, Germany; 5 DZHK (German Centre for Cardiovascular Research), Partner Site Munich Heart Alliance, Munich, Germany

**Keywords:** Exercise addiction, Mental health, Alpine sports, Mountaineering, Behavioral addiction

## Abstract

Common knowledge implies that individuals engaging in outdoor sports and especially in regular and extreme mountaineering are exceptionally healthy and hardened. Physical activity in outdoor environments has a positive effect on physical and mental health. However, regular and/or extreme mountaineering might share similarities with behavioural addictions and could thus also have a negative impact on health. In this cross-sectional web-based questionnaire study, we collected data on exercise and mountaineering addiction (Exercise Addiction Inventory; original and adapted version for mountaineering; Exercise Dependence Scale adapted version for mountaineering). Further surveyed parameters included mountaineering habits, Risk-Taking Inventory, Sensation-Seeking/Emotion Regulation/Agency Scale (SEAS), resilience, self-perceived stress, physical activity in metabolic units and mental health. Comparisons were performed between individuals with symptoms of addiction to mountaineering (MA) and individuals without symptoms of addiction to mountaineering or sports in general (CO) using non-parametric analyses. We analysed data from 335 participants, *n* = 88 thereof with addiction to mountaineering (MA) and *n* = 247 control participants (CO). The MA group scored significantly higher with regards to self-perceived stress (*p* < 0.001) and included a significantly higher number of individuals affected by symptoms of depression (*p* < 0.001), symptoms of anxiety (*p* < 0.001), symptoms of eating disorders (*p* < 0.001), alcohol abuse or dependence (*p* < 0.001), illicit drug abuse (*p* = 0.050), or current and history of psychiatric disorders (*p* < 0.001). Individuals with MA showed higher values in all SEAS subscales as well as increased risk-taking (*p* < 0.001). Regular and extreme mountaineering can display features of a behavioural addiction and is associated with psychiatric disorders. Behavioural addiction in mountaineering is associated with higher levels of sensation-seeking, emotion regulation, and agency, as well as increased risk-taking.

**Supplementary Information:**

The online version contains supplementary material available at 10.1007/s00406-022-01476-8.

## Introduction

Aphorisms like "The mountains are calling, and I must go" by the famous mountaineer John Muir encapsulate how individuals are attracted to outdoor sports. Several studies have shown that physical activity, especially in outdoor environments, has a positive effect on physical and mental health [1–3]. Regular exercise enhances general physical well-being, mood, and reduces anxiety, whereas reduced physical activity is affiliated with chronic disease [4]. Furthermore, researchers have reported that in healthy individual taking part in high-risk sports and mountaineering could have a positive impact on everyday functioning and self-esteem, as well as emotion regulation and agency [5], despite the high risk of injury or even death [6]. But there are also aspects of excessive exercise which go beyond salutogenic effects and might actually share similarities with psychiatric disorders (e.g. behavioural addictions, depressive symptoms in overtraining, obsessive–compulsive disorder) [4].

The relationship between behavioural addiction and mountaineering has not been previously addressed in the literature. Moreover, no clinical criteria are available to diagnose behavioural addiction in DSM-V or ICD-10/11, except for gambling disorder. Behavioural addiction implies a compulsion to engage in a rewarding non-substance-related behaviour. The ICD-10 codes this form of addiction in F63.—as “abnormal habitude and impulse control disorder” [7], in ICD-11, there is the category of “Disorders due to addictive behaviours” which will include “gambling and gaming”, “impulse control disorder” as well as “other” [8]. This labelling is highly controversial in the scientific community and many advocate the inclusion of further behavioural addictions, such as exercise addiction, into the classification systems [9]. Nevertheless, there are some studies evaluating the categorisation of excessive physical activity as a behavioural addiction. Exercise addiction involves performing excessive amounts of exercise to the detriment of physical health, spending too much time exercising resulting in functional impairments in the personal sphere of life, and exercising regardless of physical injury [10]. These symptoms also occur as a diagnostic criterion of anorexia nervosa, a disorder which shows several aspects of a behavioural addiction [11]. A recent systematic review summarising studies with individuals at risk for exercise addiction showed increased rates of a variety of mental disorders, including eating disorders, depression and anxiety symptoms [12]. Literature indicates that there are individuals with mental conditions in consequence of their exercise habits [13]. The WHO provides no recommendations for upper limits in terms of intensity, frequency, and duration of physical activity, which neglects an insufficiently studied health problem: whilst most people exercise too little, some exercise too much and even display addiction-like behaviour [14]. Excessive physical exercise can lead to so called “overtraining syndrome” which is patho-physiologically and symptomatically linked to depression [15] and other addictive behaviours like Internet addiction, gambling, and workaholism [16]. There is some evidence of the coexistence between exercise addiction and nicotine, alcohol, and drug abuse [17], which was confuted by other studies [18]. This shows that the literature on this topic is still controversial.

The primary aim of our study was to explore whether behavioural addiction to mountaineering exists and if there is an association with stress-related psychiatric disorders (e.g. co-occurrence with other addictions or depressive and anxiety symptoms). Its secondary aim was to evaluate resilience levels, sensation-seeking, emotion regulation, agency, and risk-taking behaviour in alpine sports in individuals with and without addiction to mountaineering.

## Methods

### Study design

This is a cross-sectional study. The anonymous web-based questionnaire was sent out as a link through email distributors of the Austrian/German Alpine Clubs, Austrian/German Alpine Mountain Guides Association, posted on the respective associations websites and promoted via social media in German-speaking (Austrian, German, Italian, Swiss) Facebook groups addressing mountaineering topics. Informed consent and data protection statement were provided on the first page of the online questionnaire. Study recruitment was conducted over a 3-month period during fall/winter 2019–2020. The survey contained mainly standardised and previously validated questionnaires. The study protocol was authorised and approved by the ethics commission of the Medical University of Innsbruck (Ethic commission number: 1191/2019).

### Participants

The study addressed German-speaking individuals who self-identified as regular or extreme mountaineers. Mountaineering was defined in the participation call as all alpine activities covering a distance and gaining vertical metres with the aim to reach a summit or another prominent destination. This included alpine climbing, trekking, high altitude mountaineering, trail running, and ski mountaineering, whilst excluding sport climbing in the climbing garden and alpine pasture hikes. We did not define the specifiers “regular” or “extreme” in the study call since there is no universally accepted definition. Most mountaineers would agree that “regular” is equal to one or more mountaineering activities per week whilst the term “extreme” is used for mountaineering activities outside of signposted or secured areas as well as expedition mountaineering involving high technical demands and high risk. Inclusion criteria were self-reported participation in regular or extreme mountaineering (by the provided definition), fluency in German and age over 18 years. No exclusion criteria were given. Due to the anonymity of the questionnaire, we did not distinguish professional athletes from amateur athletes. Specific aspects of participants´ mountaineering behaviour were then surveyed in the questionnaire.

### Measures

Sociodemographic parameters (e.g. age, sex, BMI, marital status) and mountaineering-related factors (i.e. vertical height in m/week, number of peaks climbed per week, age participants started mountaineering), as well as current or former somatic or psychiatric disorders with special focus on addiction, family history of addiction, and injuries related to mountaineering were surveyed with self-constructed questions (the full questions are given in the supplemental material 1 section).

Exercise addiction was measured with the German Exercise Dependence Scale (EDS, [19]) and the German Exercise Addiction Inventory (EAI, [20]). For the EAI, two versions were used: the original version and an adapted version to address mountaineering specifically (EAI-M). The EAI is a short 6-item instrument to identify exercise addiction by addressing salience, mood modification, tolerance, withdrawal symptoms, conflict, and relapse [21]. For the EDS, an adapted version to address mountaineering specifically (EDS-M) was used. The EDS and the EAI were adapted to mountaineering by substituting the words “sport”/“training” with “mountaineering”. The EDS operationalises exercise dependence according to the DSM-IV including criteria for substance dependence like tolerance, withdrawal, intention, lack of control, time, reductions in other activities or continuance [22]. EAI and EAI-M scores of 24 were used as cut-off values [23]. For EDS the cut-off value was 77 [19]. The EAI and the EDS are two established questionnaires designed to measure addictive characteristics in exercise behaviour and we decided to use both in the current study to capture the construct in a more conclusive way, since there is no “gold standard” to diagnose exercise addiction to date.

Mental health was assessed using subscales of the German version of Patient Health Questionnaire (PHQ-4 [24] screening for depressive symptoms and anxiety) and the sections focussing on eating disorder and alcohol [25]. Positive screening for a possible eating disorder was based on PHQ [26] scorings (module on eating disorders) as well as the body mass index < 18 kg/m^2^. The BMI criterion was introduced as a surrogate because the PHQ eating disorder module does not assess anorexia. Participants were scored as “current psychiatric disorder positive” if they scored positive in any of the used PHQ modules or self-reported a psychiatric condition not screened for in the PHQ modules used.

Resilience was measured using the German “Resilienzskala—RS-13”, which is a short version of the original RS-25. The minimum score is 13 and maximum score is 91, with values between 65 and 73 representing moderate resilience, values below low resilience, and values above high resilience [27]. To assess the willingness to take risks as well as sensation-seeking during mountaineering, we used the German version of the Sensation-Seeking/Emotion Regulation/Agency Scale (G-SEAS) and the Risk-Taking Inventory (G-RTI) [28, 29]. The G-SEAS measures the need for sensation, difficulty with emotion regulation, and lack of agency. This is based on a study showing that participants in high-risk activities have difficulty with a reduced sense of agency in aspects of their life and emotion regulation, yet improving these qualities through high-risk sport activities [29]. The RTI measures deliberate risk-taking and precautionary behaviours in high-risk sports [28]. Self-perceived stress was measured using the German version of the Perceived Stress Scale (PSS-4) [30]. Physical activity was assessed using the standardised Global Physical Activity Questionnaire (GPAQ) provided by the WHO [31]. Physical activity was calculated using metabolic equivalents (METs) as a unit for energy use. This questionnaire was adapted with a version specifically for physical activity during mountaineering.

The questionnaires were presented in the following order: sociodemographic, self-constructed questions on physical and mental health, self-constructed questions on addiction, self-constructed questions related to mountaineering behaviour, RS-13, EAI-M, EAI, EDS-M, SEAS, RTI, PHQ, PSS, GPAQ.

### Statistical methods

A total of 602 German-speaking mountaineers opened the link, 222 thereof were excluded due to incomplete data until the EDS-M. Additionally, individuals with addiction to physical activity only according to the EAI (not specifically to mountaineering) were excluded from the present analysis (PA, *n* = 45). Hence, 335 were included in the final analysis. The Exercise Addiction Inventory (EAI) and the specific version adapted for mountaineering (EAI-M) was used for assigning individuals either to the group of individuals who showed behavioural addiction to mountaineering (MA, *n* = 88) or individuals with no behavioural addiction to mountaineering or physical activity in general (*n* = 247, CO).

Prior to the analysis, metric variables were checked for deviations from normality by assessing their skewness, considering values > 0.5 or < − 0.5 as deviations from a symmetric distribution requiring non-parametric testing. The focus of the analysis was placed on a comparison of the group of individuals who showed behavioural addiction to mountaineering (MA) and individuals with no behavioural addiction to mountaineering or physical activity in general (CO). These comparisons were performed by means of *t *Test, Mann–Whitney *U* Test or Chi-Square Test, depending on the variable type (normally distributed, non-normally distributed metric variables, or categorical variables, respectively). Apart from comparisons with respect to socio-demographics and mountaineering-related aspects, we were mainly interested in investigating group differences regarding clinical features, addictive disorders, salutogenic and pathogenic aspects, as well as physical activity, using the same tests as above for this purpose. As the two groups differed significantly in their age, marital status, and employment status, we also performed group comparisons with adjustment for these three variables. This was done by means of analysis of covariance for metric dependent variables, by logistic regression for binary and by ordinal regression for ordinal dependent variables (Supplemental Material 2). A correlation analysis between EDS-M and EAI-M was also conducted to demonstrate the concurrent validity of the two measures. All statistical analyses were conducted using SPSS, version 26.

## Results

### Socio-demographics and mountaineering-related features

Participants with behavioural addiction to mountaineering (MA (mountaineering addiction), *n* = 88) as defined by the Exercise Addiction Inventory (EAI-M, adapted version for mountaineering) were compared to individuals with no behavioural addiction to mountaineering or physical activity in general (CO (controls), *n* = 247). Scorings on the EAI-M and the Exercise Dependence Scale adapted for mountaineering (EDS-M) correlated with each other over both groups i.e. MA and CO (Spearman’s rank order correlations, *r* = 0.760 *p* < 0.001). When component scorings of EDS-M were analysed, the MA group showed significant higher values in all parameters of behavioural addiction (withdrawal effects, continuance, tolerance, lack of control, reduction in other activities, time, intention effect) compared to CO (Mann–Whitney *U* Test, all *p* < 0.001). Table 1 shows the sociodemographic characteristics divided by groups. MA had a lower mean age (Mann–Whitney *U* Test, *p* < 0.001) and a slightly lower BMI (Mann–Whitney *U* Test, *p* = 0.031; however, significance is lost after adjustment for age, marital status and employment, general linear model, *p* = 0.300). Within the group of MA, there was a higher percentage of single people (77.3% compared to 62.3% in CO group). Objective parameters related to mountaineering frequency and habits differed significantly between MA and CO (Table 1). Individuals in the MA group climbed a higher number of peaks per week (Chi-Square Test, *p* < 0.001), were more likely to go mountaineering during off-season (Chi-Square Test, *p* < 0.001), and had fewer ‘mountaineering-free’ days per weeks (Chi-Square Test, *p* < 0.001).

**Table 1:** Sociodemographic data

Variable	MA (*n* = 88)	CO (*n* = 247)	Comparison test statistics	*df*	*p *value
Age in years^a^	31.1 ± 10.0	39.0 ± 13.2	*Z* = − 4.81^c^		< 0.001
BMI^a^	22.3 ± 2.6	23.1 ± 2.6	*Z* = − 2.16^c^		0.031
Gender^b^				1	0.697
Male	55 (62.5%)	152 (61.5%)			
Female	33 (37.5%)	93 (38.5%)			
Marital status^b^			*χ*^2^ = 8.69^d^	2	0.013
Single	68 (77.3%)	154 (62.3%)		–	–
Married/partnership	19 (21.6%)	73 (29.6%)		–	–
Divorced/widowed	1 (1.1%)	20 (8.1%)		–	–
Employment^b^			*χ*^2^ = 8.39^d^	2	0.015
Full-/part-time employment	53 (60.2%)	185 (74.9%)		–	–
Apprenticeship/study/vocational training	30 (34.1%)	47 (19.0%)		–	–
Other	5 (5.7%)	15 (6.1%)		–	–
Age participants started mountaineering^b^			*χ*^2^ = 3.30^d^	2	0.192
< 10 years	33 (37.5%)	69 (28.0%)		–	–
10–30 years	47 (53.4%)	148 (60.2%)		–	–
> 30 years	7 (8.0%)	29 (10.6%)		–	–
Climbed peaks/week^b^			*χ*^2^ = 31.48^d^	2	< 0.001
0–1	20 (22.7%)	137 (55.5%)		–	–
2–3	57 (64.8%)	80 (32.4%)		–	–
> 3	11 (12.5%)	30 (12.1%)		–	–
Vertical metres/week^b^			*χ*^2^ = 42.84^d^	2	< 0.001
< 1000	6 (6.8%)	112 (45.3%)		–	–
1000–3000	66 (75.0%)	103 (41.7%)		–	–
> 3000	16 (18.2%)	32 (13.0%)		–	–
Mountaineering during off-season^b^			*χ*^2^ = 13.21^d^	1	< 0.001
Yes	74 (84.1%)	156 (63.2%)		–	–
No	14 (15.9%)	91 (36.8%)		–	–
Times mountaineering > 5000 m sea level			*χ*^2^ = 5.49^d^	3	0.139
0	49 (55.7%)	168 (68.0%)		–	–
1–3	23 (26.1%)	54 (21.9%)		–	–
4–10	12 (13.6%)	19 (7.7%)		–	–
> 10	4 (4.5%)	6 (2.4%)		–	–
Mountaineering free days/week^b^			*χ*^2^ = 22.71^d^	2	< 0.001
5–7	25 (28.4%)	143 (57.9%)		–	–
3–4	42 (47.7%)	72 (29.1%)		–	–
1–2	21 (23.9%)	32 (13.0%)		–	–

Results of socio-demographics as well as factors related to mountaineering activity

*MA* addiction to mountaineering, *CO* no addiction to mountaineering or general physical activity

^a^Mean ± standard deviation

^b^Absolute number (column per cent)

^c^Mann–Whitney *U* Test

^d^Chi-Square Test

### Mountaineering addiction is associated with psychiatric disorders

Individuals in the MA group showed higher point prevalence of psychiatric disorders than CO (Table 2). Specifically, MA had significantly higher values than CO regarding self-perceived stress (Mann–Whitney *U* Test, *p* < 0.001), symptoms of eating disorders (Chi-Square Test, *p* < 0.001), symptoms of depression (Chi-Square Test, *p* < 0.001), symptoms of anxiety (Chi-Square Test, *p* < 0.001) and current psychiatric disorders, whilst there was no significant difference in resilience. Individuals with MA more often self-reported a positive history of a pre-existing psychiatric disorder (depression /depressive symptoms (*n* = 23), anxiety and panic disorders (*n* = 4), eating disorder (*n* = 9) and “other” (*n* = 6) including diagnoses, such as obsessive–compulsive disorder or attention-deficit hyperactivity disorder Chi-Square Test, *p* < 0.001). No difference was found for a history of somatic conditions (self-report: musculoskeletal disorders (*n* = 22), cardiovascular (*n* = 6), pulmonal (*n* = 13), neurological (*n* = 4) and “other” (*n* = 25) including diagnoses, such as dermatitis, hearing loss, allergies), or mountaineering-related physical injuries. All significances are retained when adjusting for age, marital status, and employment (Supplemental Material 2, Table 2, Chi-Square Test).

**Table 2:** Clinical features, resilience, and self-perceived stress

	MA (*n* = 88)	CO (*n* = 247)	Comparison test statistics	*df*	*p* value
Depressive symptoms^b^	21.6% (19)	7.7% (19)	*χ*^2^ = 12.46 ^d^	1	< 0.001
Anxiety symptoms^b^	20.5% (18)	8.5% (21)	*χ*^2^ = 9.01 ^d^	1	< 0.003
Self-perceived stress^a^	10.2 ± 2.9	8.8 ± 2.7	*Z* = − 3.95 ^c^	1	< 0.001
Resilience^a^	74.9 ± 10.8	74.1 ± 9.3	*Z* = − 1.09 ^c^	1	0.273
Symptoms of eating disorder^b^	22.1% (19)	5.7% (14)	*χ*^2^ = 18.52 ^d^	1	< 0.001
Current psychiatric disorder^b^	52.3% (46)	27.5% (68)	*χ*^2^ = 17.69 ^d^	1	< 0.001
History of psychiatric disorder^b^	19.3% (17)	5.3% (13)	*χ*^2^ = 15.72 ^d^	1	< 0.001
Injuries related to mountaineering^b^	54.5% (48)	49% (121)	*χ*^2^ = 0.802 ^d^	1	0.371
Current somatic disorders^b^	17.0% (15)	16.6% (41)	*χ*^2^ = 0.009 ^d^	1	0.923

Comparison of MA and CO in clinical features, resilience, and self-perceived stress

*MA* addiction to mountaineering, *CO* no addiction to mountaineering or general physical activity

^a^Mean ± standard deviation

^b^Column per cent (absolute number)

^c^Mann–Whitney *U* Test

^d^Chi-Square Test

### Mountaineering addiction is associated with other addictive disorders

The MA group showed a higher prevalence of other addiction disorders (Table 3). Specifically, individuals in the MA group more frequently reported a history of addictive disorders (Chi-Square Test, *p* < 0.001) as well as currently active addictive disorders (Chi-Square Test, *p* < 0.001) more frequently than CO. Symptoms of alcohol abuse or dependency (Chi-Square Test, *p* < 0.001) as well as illicit drug use (Chi-Square Test, *p* = 0.05) was more frequent in MA. No difference was found concerning smoking. All significances except those for illicit drug use and current addiction disorder are retained after adjustment for age, marital status, and employment (Supplemental Material 2, Table 3, Chi-Square Test).

**Table 3:** Addictive behaviour and disorders

	MA (*n* = 88)	CO (*n* = 247)	Comparison test statistics	*df*	*p* value
Symptoms of alcohol abuse or dependence	26.1% (23)	11.7% (29)	*χ*^2^ = 10.25	1	< 0.001
Nicotine use	9.1% (8)	6.1% (15)	*χ*^2^ = 0.92	1	0.336
Illicit drug use (mostly marihuana)	10.2% (9)	4.5% (11)	*χ*^2^ = 3.85	1	0.050
Current addiction disorder (substance or behavioural addiction)	5.7% (5)	1.2% (3)	*χ*^2^ = 5.555	1	0.018
History of addiction disorder	10.2% (9)	3.2% (8)	*χ*^2^ = 6.578	1	0.010
Positive family history of addiction disorder	13.6% (12)	7.7% (19)	*χ*^2^ = 2.73	1	0.098

Comparison of MA and CO regarding addictive behaviour and disorders

Chi-Square Test was used for analysis

Results are given as colum per cent (absolute number)

*MA* addiction to mountaineering, *CO* no addiction to mountaineering or general physical activity

### The Mountaineering addiction group shows higher levels of intensive physical activity

Individuals with MA scored higher in general physical activity (Mann–Whitney *U* Test, *p* = 0.017) as well as in physical activity related to mountaineering (Mann–Whitney *U* Test, *p* < 0.001, Table 4). Notably, this difference was due to higher values only for intensive physical activity but not for moderate values of metabolic equivalents (METS) which were comparable between the groups. All significances are kept when adjusting for age, marital status, and employment (Supplemental Material 2, Table 4, Mann–Whitney *U* Test).

**Table 4:** Physical activity, sensation-seeking, emotion regulation, agency, risk-taking and cautiousness

Variable	MA	CO	Comparison test statistics	*p* value
P total^a^	15,240 ± 8794	14,102 ± 10,429	*Z* = – 2.392	0.017
P intensive^a^	8716 ± 7512	7215 ± 8116	*Z* = – 3.423	< 0.001
P moderate^a^	4221 ± 3686	4307 ± 4254	*Z* = – .639	0.523
M total^a^	5797 ± 5616	3357 ± 4111	*Z* = – 4.943	< 0.001
M intensive^a^	4670 ± 5266	2455 ± 3445	*Z* = – 4.966	< 0.001
M moderate^a^	1017 ± 1419	875 ± 1492	*Z* = – 1.054	0.292
Sensation	22.8 ± 3.6	18.0 ± 4.7	*Z* = – 8.104	< 0.001
Regulation	23.6 ± 3.8	19.5 ± 5.0	*Z* = – 6.914	< 0.001
Agency	36.8 ± 4.9	34.1 ± 4.9	*Z* = – 5.005	< 0.001
Risk-taking	8.2 ± 3.6	5.4 ± 2.4	*Z* = – 6.639	< 0.001
Cautiousness	17.1 ± 3.0	17.0 ± 2.8	*Z* = – 0.479	0.632

Comparison of MA and CO regarding physical activity, sensation-seeking, emotion regulation, agency, risk-taking and cautiousness

Results of GPAQ (General Physical Activity Questionnaire) the G-SEAS (German Sensation-Seeking Emotion Regulation and Agency Scale) and G-RTI (German Risk-Taking Inventory)

Physical activity is given in (MET minutes/week)

Mean ± standard deviation is given

Mann–Whitney *U* Test was used for comparison

*MA* addiction to mountaineering, *CO* no addiction to mountaineering or general physical activity, *P* general physical activity, *M* mountaineering, *MET* metabolic units

^a^7 missings

### Mountaineering addiction is associated with increased sensation-seeking and risk-taking

Individuals with MA showed higher values for the use of on the G-SEAS scale compared to CO in all assessed domains which are sensation-seeking (Mann–Whitney *U* Test, *p* < 0.001), emotion regulation (Mann–Whitney *U* Test, *p* < 0.001), agency (Mann–Whitney *U* Test, *p* < 0.001). Despite, higher values for risk-taking were found (Mann–Whitney *U* Test, *p* < 0.001; Table 4). No difference between the groups was found for cautiousness (Table 4).

## Discussion

The key finding of our study is that regular and extreme mountaineering can show characteristic properties of behavioural addictions. Whilst the majority of individuals who performed regular and/or extreme mountaineering do not show behavioural addiction, there is a subgroup of mountaineers who are vulnerable to addiction concerning alpine sports. The problem of addressing behavioural addiction starts with inconsistencies in terminology and in assessment tools. The two most accepted assessment tools for assessing addictive features of physical exercise are the EAI and EDS which we also used in the current study. Both show similar results in our study [14].

We found an association of behavioural addiction to mountaineering with symptoms of depression and anxiety as well as with a history of psychiatric disorders and with higher levels of mental stress. This is at first glance surprising since outdoor physical activity is known to promote mental health [3]. Previous literature has shown a relationship between behavioural addiction in general and depressive and anxiety disorders [32]. It has been shown that excessive training in amateur endurance cyclists is associated with reduced quality of life, worse sleep and increased levels of anxiety [33], which may lead to withdrawal and uncontrolled behaviour [15]. Addictive disorders in general show a high comorbidity with anxiety and mood disorders [34]. On the one side, exercise has anti-depressive and anxiolytic properties in people with depression and is therefore used as a coping mechanism and as a therapy for psychiatric diseases [35]. On the other side, there is a subgroup of people which loses control over their exercise behaviour and continues exercising despite negative mental and physical consequences [12]. With the current data, it is not possible to determine the direction of the relation between psychiatric symptoms and excessive mountaineering. Psychological proneness to addiction could lead to excessive mountaineering, or psychiatric problems could occur as a result of the burden of exercise addiction [12].

Additionally, individuals with addiction to mountaineering show higher numbers of further addictive disorders, such as alcohol, illicit drug use and self-reported diagnosis of current or history of addictive disorders. There are common risk factors between the co-occurrence of behavioural dependencies and substance use disorders in the same individual as shown in the literature [17]. These findings are, however, not unequivocal [18]. Some people are more prone to addictive behaviours, regardless of whether these involve substances or problematic activities [32]. Multiple studies show a relation between childhood trauma and addiction, such as drug addiction [36], gambling disorder [37] and substance use amongst adolescents [38]. Furthermore, a genetic and epigenetic link is possible [39].

Addiction to mountaineering is associated with higher levels of sensation-seeking, emotion regulation and agency as well as increased risk-taking. Those individuals with addictive behaviour related to mountaineering showed higher levels of sensation-seeking, emotional regulation, and agency during mountaineering. This might help to explain the role that excessive mountaineering plays in these individuals’ lives: problematic exercise has been shown to be associated with both impulsivity and compulsivity, which could explain the higher values of the SEAS scale found in our sample [40]. Exercise dependence negatively correlates with the personal resources trait, state self-control, and self-concordance [41]. The increased risk-taking behaviour can also interact with one’s regular life, leading to negative effects on mental health [29, 42] and could also reflect personality traits of individuals with addiction to mountaineering. Sensation seeking and risk-taking have been shown to be associated with addictive behaviour [43].

In the current study, we also found an association between addiction to mountaineering and symptoms of eating disorders. This comorbidity might stem from the fact that both eating disorders and excessive exercise share some pathophysiological aspects of behavioural addiction [44]. Excessive exercise is frequently associated with eating disorders and may evolve into exercise addiction [45]. We found an association also with other addictive disorders which is in line with research showing that addictions often come in clusters [46]. Whilst for some addictions (e.g. smoking, alcohol or drug use), this is easier to understand considering a common pathophysiology and genetic component [47], addiction to exercise and mountaineering in particular is a different story. Since outdoor physical activity such as mountaineering is generally considered to promote mental health, its co-occurrence with addictions with a well-known negative impact on mental and physical health is nevertheless to some extent surprising. The similar levels of resilience found in MA and controls could be a hint towards the health-related aspects of mountaineering even when it is performed excessively which differentiates it from other behavioural addictions. Like all other addictions, exercise addiction may reflect an escape from a hardship along with an accessible way to overcome negative criticism, because exercise itself is a positive and socially valued behaviour [14]. It is also possible that individuals with psychiatric disorders or symptoms use regular and extreme mountaineering as part of a “self-therapy” to partially overcome their symptoms.

### Limitations

The extent and quality of the evidence are explorative and bear several limitations. The main limitation is related to the selection of the current sample: including only German-speaking individuals who self-classified as regular or extreme mountaineers enabled as to include a very high number of individuals with addiction to mountaineering. On the other hand, it must be kept in mind that this is of course a highly selective sample, and results cannot be applied to the general population, especially since we did not assess whether individuals were professional athletes. The distinction between mountaineering addiction and addiction to general physical activity is partly artificial, since in practice, both categories range on a spectrum rather than in two different classes. Furthermore, analysis relies on self-evaluated questionnaires and self-assessed ratings and the way we operationalised eating disorders is not ideal to evaluate diagnoses. There is also a high number of excluded participants, due to incomplete data. When interpreting the results, caution needs to be taken since causal relationships were not investigated.

## Conclusion

We define a group of individuals with addiction specifically to mountaineering and the co-occurrence with other addictions or depressive and anxiety symptoms. Even though physical activity outdoors especially in natural environments are generally considered healthy, it can lead to negative effects on mental health if taken to a level of addictive behaviour, involving features, such as mood modification, tolerance, withdrawal symptoms, conflict, and relapse. Furthermore, addiction to mountaineering is associated with higher levels of sensation-seeking, emotion regulation, and agency, as well as increased risk-taking. Future studies are needed to better understand whether mental disorders are a succession of excessive mountaineering, or mountaineering is used as a form of “self- therapy” to reduce mental symptom burden.

## Supplementary Information

Below is the link to the electronic supplementary material.

Supplementary file1 (DOCX 20 KB)

Supplementary file2 (DOCX 39 KB)
