# Supplementary material for: Why do we climb mountains? An exploration of features of behavioural addiction in mountaineering and the association with stress-related psychiatric disorders
Source: Eur Arch Psychiatry Clin Neurosci. 2022 Aug 18;273(3):639–47. doi: 10.1007/s00406-022-01476-8 (PMC10085896; doi:10.1007/s00406-022-01476-8)
Supplement: Supplementary file 1 — Supplementary file1 (DOCX 20 KB) [file 406_2022_1476_MOESM1_ESM.docx]

**Supplemental Material 1**

Questionnaires used in the survey

**Personal information**

- Age: in years
- Sex: male, female, diverse
- Profession: free text field
- Education and occupation with working time: full day, half day, unemployed, retired, housewife/ -husband, sick leave, apprenticeship, studying, other
- Income of the household per month: <1000€, 1000-2000€, 2000-5000€, >5000€
- Marital status: single, married/in partnership, widowed, divorced
- Children: yes, no, if yes: number
- Size (in meters) and weight (in kilograms)
- Intake of medication: yes, no, if yes: list of medication
- Alcohol consumption: wine, beer, spirit in glasses/week
- Nicotine consumption: yes, no, if yes: cigarettes/day
- Other drugs consumption (i.e., cannabis): yes, no, if yes: dose/week
- Former and current somatic disorders: free text field
- Former and current psychiatric disorders: free text field
- Current addictive disorders: yes, no, list of diagnoses and who made the diagnose (doctor or other mental health professional, self-diagnosed, another person)
- Addictive disorders in family: yes, no, list of persons (mother, father, brother, sister, grandparents, other) with diagnose and who made the diagnose (doctor or other mental health professional, self-diagnosed, another person)
- Former sport injuries: yes, no, if yes: list of injuries, date of injuries, persisting problems fromthe injury if any
- Former mountaineering injuries: yes, no, if yes: list of injuries, date of injuries, persisting problems fromthe injury if any
- Vertical meters climbed per week during season: <1000, 1000-3000, >3000
- Hours of mountaineering spent per week during season: <2, 2-5, >5
- Summits or prominent points reached per week during season: <1, 1-3, >3
- Mountaineering during off-season (e.g. skiing in summer or trailrunning in the snow): yes, no
- Travelling abroad with primary aimto go mountaineering: yes, no
- Number of summits >5000 hight metres reached until date: <1, 1-3, 4-10, >10
- Number of summits >7000 hight metres reached until date: <1, 1-3, 4-10, >10
- Mountaineering free days per week: 4-7, 2-3, 0-1
- Main sport during summer season: free text field
- Main sport during winter season: free text field
- Mountaineering experience in childhood: yes, no
- Age when starting mountaineering: in years
- Special diet: yes, no, if yes: which (free text field)
- Food intolerance: yes, no, if yes: which

**Scale of resilience** (RS-13)

**Exercise Addiction Inventory** (EAI)

**Exercise Addiction Inventory adapted for mountaineering** (EAI-M): the word “training” was substituted by the word “mountaineering”

**Exercise Dependence Scale adapted for mountaineering** (EDS-M): the word “sport” was substituted by the word “mountaineering”

**Sensation Seeking, Emotion Regulation, Agency Scale (**SEAS): version during mountaineering

**Risk Taking Inventory** (RTI)

**Patient Health Questionnaire screening questions for depression and anxiety (**PHQ-4)

**Perceived Stress Scale** (PSS-4)

**Global Physical Activity Questionnaire (**GPAQ)
