# Supplementary material for: Why do we climb mountains? An exploration of features of behavioural addiction in mountaineering and the association with stress-related psychiatric disorders
Source: Eur Arch Psychiatry Clin Neurosci. 2022 Aug 18;273(3):639–47. doi: 10.1007/s00406-022-01476-8 (PMC10085896; doi:10.1007/s00406-022-01476-8)
Supplement: Supplementary file 2 — Supplementary file2 (DOCX 39 KB) [file 406_2022_1476_MOESM2_ESM.docx]

**Supplemental material 2 - Adjustment for age, marital status, and employment**

**Table 1** Sociodemographic data

| **Variable** | **MA (n=88)** | **CO (n=247)** | **Comparison Test statistics** | **Df** | **p-value** | **Adjustment for age, marital status, and employment^e^** |
| --- | --- | --- | --- | --- | --- | --- |
| Age in years^a^ | 31.1 ± 10.0 | 39.0 ± 13.2 | Z = -4.81^c^ |  | <0.001 |  |
| BMI^a^ | 22.3 ± 2.6 | 23.1 ± 2.6 | Z = -2.16 ^c^ |  | 0.031 | F= 1.08  p=0.300 |
| Gender^b^ |  |  |  | 1 | 0.697 |  |
| Male | 55 (62.5%) | 152 (61.5%) |  |  |  |  |
| Female | 33 (37.5%) | 93 (38.5%) |  |  |  |  |
| Marital status^b^ |  |  | χ² = 8.69 ^d^ | 2 | 0.013 |  |
| Single | 68 (77.3%) | 154 (62.3%) |  | - | - |  |
| Married/partnership | 19 (21.6%) | 73 (29.6%) |  | - | - |  |
| Divorced/  widowed | 1 (1.1%) | 20 (8.1%) |  | - | - |  |
| Employment^b^ |  |  | χ² = 8.39 ^d^ | 2 | 0.015 |  |
| Full-/part-time  employment | 53 (60.2%) | 185 (74.9%) |  | - | - |  |
| Apprenticeship/study/  vocational training | 30 (34.1%) | 47 (19.0%) |  | - | - |  |
| Other | 5 (5.7%) | 15 (6.1%) |  | - | - |  |
| Age participants started mountaineering^b^ |  |  | χ² = 3.30 ^d^ | 2 | 0.192 |  |
| < 10 years | 33 (37.5%) | 69 (28.0%) |  | - | - |  |
| 10 – 30 years | 47 (53.4%) | 148 (60.2%) |  | - | - |  |
| > 30 years | 7 (8.0%) | 29 (10.6%) |  | - | - |  |
| Climbed peaks/ week^b^ |  |  | χ² = 31.48 ^d^ | 2 | <0.001 | χ² = 13.07  p<0.001 |
| 0-1 | 20 (22.7%) | 137 (55.5%) |  | - | - |  |
| 2-3 | 57 (64.8%) | 80 (32.4%) |  | - | - |  |
| >3 | 11 (12.5%) | 30 (12.1%) |  | - | - |  |
| Vertical meters/ week^b^ |  |  | χ² = 42.84 ^d^ | 2 | <0.001 | χ² = 24.49  p<0.001 |
| <1000 | 6 (6.8%) | 112 (45.3%) |  | - | - |  |
| 1000-3000 | 66 (75.0%) | 103 (41.7%) |  | - | - |  |
| >3000 | 16 (18.2%) | 32 (13.0%) |  | - | - |  |
| Mountaineering during off-season^b^ |  |  | χ² = 13.21^d^ | 1 | <0.001 | χ² = 11.90  p<0.001 |
| Yes | 74 (84.1%) | 156 (63.2%) |  | - | - |  |
| No | 14 (15.9%) | 91 (36.8%) |  | - | - |  |
| Times Mountaineering >5000m sea level |  |  | χ² = 5.49 ^d^ | 3 | 0.139 | χ² = 9.76  p=0.002 |
| 0 | 49 (55.7%) | 168 (68.0%) |  | - | - |  |
| 1-3 | 23 (26.1%) | 54 (21.9%) |  | - | - |  |
| 4-10 | 12 (13.6%) | 19 (7.7%) |  | - | - |  |
| >10 | 4 (4.5%) | 6 (2.4%) |  | - | - |  |
| Mountaineering free days/ week^b^ |  |  | χ² = 22.71^d^ | 2 | <0.001 | χ² = 19.06  p<0.001 |
| 5-7 | 25 (28.4%) | 143 (57.9%) |  | - | - |  |
| 3-4 | 42 (47.7%) | 72 (29.1%) |  | - | - |  |
| 1-2 | 21 (23.9%) | 32 (13.0%) |  | - | - |  |

*Results of sociodemographics as well as factors related to mountaineering activity*

^a^ Mean ± standard deviation
^b^ Column percent (absolute number)

^c^ Mann Whitney U Test
^d^ Chi Square Test

^e^ Adjustment by means of general linear models (F) or ordinal logistic regression (χ²).

Abbreviations: MA: Addiction to mountaineering, CO: No addiction to mountaineering or general physical activity

**Table 2** Clinical features, resilience, and self-perceived stress

|  | **MA (n=88)** | **CO (n=247)** | **Comparison**  **Test statistics** | **Df** | **p-value** | **Adjustment for age, marital status, and employment^e^** |
| --- | --- | --- | --- | --- | --- | --- |
| Depressive symptoms^b^ | 21.6% (19) | 7.7% (19) | χ² = 12.46 ^d^ | 1 | <0.001 | χ² = 8.24  p=0.004 |
| Anxiety symptoms^b^ | 20.5% (18) | 8.5% (21) | χ² = 9.01 ^d^ | 1 | <0.003 | χ² = 8.93  p=0.003 |
| Self-perceived stress^a^ | 10.2 ± 2.9 | 8.8 ± 2.7 | Z = -3.95 ^c^ | 1 | <0.001 | F = 17.65  p <0.001 |
| Resilience^a^ | 74.9 ± 10.8 | 74.1 ± 9.3 | Z = -1.09 ^c^ | 1 | 0.273 | F= 2.24  p = 0.135 |
| Symptoms of eating disorder^b^ | 22.1% (19) | 5.7% (14) | χ² = 18.52 ^d^ | 1 | <0.001 | χ² = 9.57  p = 0.002 |
| Current psychiatric disorder^b^ | 52.3% (46) | 27.5% (68) | χ² = 17.69 ^d^ | 1 | <0.001 | χ² = 19.30  p < 0.001 |
| History of psychiatric disorder^b^ | 19.3% (17) | 5.3% (13) | χ² = 15.72 ^d^ | 1 | <0.001 | χ² = 11.46  p = 0.001 |
| Injuries related to mountaineering^b^ | 54.5% (48) | 49% (121) | χ² = 0.802 ^d^ | 1 | 0.371 | χ² = 1.43  p = 0.232 |
| Current somatic disorders^b^ | 17.0% (15) | 16.6% (41) | χ² = 0.009 ^d^ | 1 | 0.923 | χ² = 0.400  p = 0.527 |

*Comparison of MA and CO in clinical features, resilience, and self-perceived stress*

^a^ Mean ± standard deviation
^b^ Column percent (absolute number)
^c^ Mann Whitney U Test

^d^ Chi Square Test
^e^ Adjustment by means of general linear models (F) or ordinal logistic regression (χ²).

Abbreviations: MA: Addiction to mountaineering, CO: No addiction to mountaineering or general physical activity

**Table 3** Addictive behaviour and disorders

|  | **MA (n=88)** | **CO (n=247)** | **Comparison**  **Test statistics^a^** | **Df** | **p-value** | **Adjustment for age, marital status, and employment^b^** |
| --- | --- | --- | --- | --- | --- | --- |
| Symptoms of alcohol abuse or dependence | 26.1% (23) | 11.7% (29) | χ² = 10.25 | 1 | <0.001 | χ² = 5.72  p= 0.017 |
| Nicotine use | 9.1% (8) | 6.1% (15) | χ² = 0.92 | 1 | 0.336 | χ² = 0.91 p = 0.341 |
| Illicit drug use (mostly marihuana) | 10.2% (9) | 4.5% (11) | χ² = 3.85 | 1 | 0.050 | χ² = 0.71 p = 0.401 |
| Current addiction disorder (substance or behavioural addiction) | 5.7% (5) | 1.2% (3) | χ² = 5.555 | 1 | 0.018 | χ² = 2.67  p = 0.102 |
| History of addiction disorder | 10.2% (9) | 3.2% (8) | χ² = 6.578 | 1 | 0.010 | χ² = 3.73  p = 0.053 |
| Positive family history of addiction disorder | 13.6% (12) | 7.7% (19) | χ² = 2.73 | 1 | 0.098 | χ² = 2.69  p = 0.101 |

*Comparison of MA and CO regarding addictive behaviour and disorders*

^a^Chi Square Test

^b^Adjustment by means of logistic regression.

Results are given as column percent (absolute numbers)

Abbreviations: MA: Addiction to mountaineering, CO: No addiction to mountaineering or general physical activity

**Table 4** Physical activity, sensation seeking, emotion regulation, agency, risk-taking and cautiousness

| **Variable** | **MA** | **CO** | **Comparison**  **Test statistics**^a^ | **p value** | Adjustment for age, marital status, employment^b^ |
| --- | --- | --- | --- | --- | --- |
| P total* | 15240 ± 8794 | 14102 ± 10429 | Z = -2.392 | 0.017 | F=6.30  p=0.013 |
| P intensive* | 8716 ± 7512 | 7215 ± 8116 | Z = -3.423 | <0.001 | F=12.92  p<0.001 |
| P moderate* | 4221 ± 3686 | 4307 ± 4254 | Z = -.639 | 0.523 | F = 0.27  p = 0.603 |
| M total* | 5797 ± 5616 | 3357 ± 4111 | Z = -4.943 | <0.001 | F=29.47  p<0.001 |
| M intensive* | 4670 ± 5266 | 2455 ± 3445 | Z = -4.966 | <0.001 | F=25.75  p<0.001 |
| M moderate* | 1017 ± 1419 | 875 ± 1492 | Z = -1.054 | 0.292 | F=3.31  p=0.070 |
| Sensation | 22.8 ± 3.6 | 18.0 ± 4.7 | Z = -8.104 | <0.001 | F=78.07  p<0.001 |
| Regulation | 23.6 ± 3.8 | 19.5 ± 5.0 | Z = -6.914 | <0.001 | F=45.75  p<0.001 |
| Agency | 36.8 ± 4.9 | 34.1 ± 4.9 | Z = -5.005 | <0.001 | F=14.87  p<0.001 |
| Risk | 8.2 ± 3.6 | 5.4 ± 2.4 | Z = -6.639 | <0.001 | F=61.23  p<0.001 |
| Cautiousness | 17.1 ± 3.0 | 17.0 ± 2.8 | Z = -0.479 | 0.632 | F=0.02 p=0.887 |

*Comparison of MA and CO regarding physical activity, sensation seeking, emotion regulation, agency, risk-taking and cautiousness*

^a^Mann-Whitney U Test

^b^Adjustment by means of general linear models.

Results of GPAQ (General Physical Activity Questionnaire) the G-SEAS (German Sensation Seeking Emotion Regulation and Agency Scale) and G-RTI (German Risk Taking Inventory).

Physical activity is given in (MET minutes/week).
Mean ± standard deviation is given.
Abbreviations: MA: Addiction to mountaineering, CO: No addiction to mountaineering or general physical activity, P: General physical activity, M: Mountaineering, MET: Metabolic units
*7 missings
